# Prevalence of sleep problems and habits among children in Saudi Arabia: A cross-sectional study

**DOI:** 10.15537/smj.2023.44.3.20220894

**Published:** 2023-03

**Authors:** Ahmed AlEidan, Mohammad Al-Shamrani, Mazen AlGhofaily, Najlaa AlDraiweesh, Basem AlGhamdi, Hattan AlHabshan, Summayah Kobeisy, Saleh Alharbi, Abdullah Al-Shamrani

**Affiliations:** *From the Department of Pediatrics (AlEidan, AlGhofaily), King Fahad Medical City, from the Department of Pediatrics (M. Al-Shamrani, AlDraiweesh, AlGhamdi, AlHabshan), Sulaiman AlHabib Medical Center, from the Department of Pediatrics (A. Al-Shamrani), Prince Sultan Military Medical City, from the Department of Pediatrics (A. Al-Shamrani), Al Faisal University, Riyadh, from the Department of Pediatrics (Kobeisy, Alharbi), Fakeeh Hospital, Jeddah, and from the Department of Pediatrics (Alharbi), Umm Al-Qura University, Makkah, Kingdom of Saudi Arabia.*

**Keywords:** sleep, sleep pattern, sleep disorders, Riyadh, Saudi Arabia

## Abstract

**Objectives:**

To investigate children’s sleep problems, habits, and lifestyle changes.

**Methods:**

A cross-sectional study was carried out in Riyadh, Saudi Arabia, over a period of 2 months, from August through September 2022, with parents of children aged 2-14 years after reviewing the literature and formulating a validated Google questionnaire containing 30 questions related to sleep habits, problems, and disorders.

**Results:**

In total, 585 questionnaires were included in the analysis. The sample comprised 345 (59%) males and 240 (41%) females. The mean age of patients was 7 (range: 2-14) years. Bed-time resistance was the most prevalent sleep problem (70.3%), followed by sleep-onset delay (58.1%), difficulty waking up in the morning on weekdays (41.3%), weekends (38%), and interrupted sleep (31%). An alarmingly high prevalence of hyperactivity (41.8%) and aggressive behaviour (42.2%) was noted. Co-sleeping with parents was reported in 41% of children. Night terror was reported in 20.6% and 26.5% in nightmares. Statistically significant associations were noted between screen time, snoring, and witnessed apnoea with sleep problems.

**Conclusion:**

Sleep problems are common among children in Saudi Arabia. The study sheds some light on sleep habits and practices in this age group in Saudi Arabia, such as the high prevalence of bed-time resistance and sleep-onset delay, hyperactivity, and sleep-affecting culprits such as screen time, snoring, and witnessed apnoea.

**O**ver the past quarter century, paediatric and adolescent sleep medicine has followed remarkable parallels with the evolution of health care for infants, children, and young adults globally.^1^ the scientific investigation of sleep and its disorders can be traced back to 1930, when Berger et al^2^ first described differentiation of sleep into specific and distinct states supported with what was reported by Harvey et al in 1937.^3^ A new era of medical and scientific research has emerged, focusing on physiology, pharmacology, pathophysiology, and even anatomy that are different during sleep than during the waking state.^4^ Sleep research provides the ground work and basis for the realisation that clinical evaluation and management of patients might differ during sleep compared with during waking, resulting in the emergence of clinical sleep medicine.^5^

Sleep patterns, sleep problems, and sleep disturbances are commonly used to describe sleep in the paediatric sleep literature and clinical setting. Sleep patterns refer to bed-times, wake times, sleep duration, and wake after sleep onset duration. Sleep problems and sleep disturbances are used interchangeably for various sleep abnormalities and refer to the disruption of bed-time, wake time, wake after sleep onset, abnormal behaviours during sleep, and poor sleep quality. Children and adolescents commonly present to primary care and mental health clinics with concerns of difficulty falling asleep and day-time sleepiness.^6^ Optimal sleep duration and sleep quality are necessary for maintaining good medical and emotional health of children and adolescents. Sleep problems are common in paediatric medical and mental health disorders and are known to have a bidirectional relationship.^7^ Sleep disorders are common and under-diagnosed in children and adolescents. In the United States of America, approximately 25% of children aged 5-12 years have sleep problems.^8^ Symptoms of insomnia have been reported in 19% of children and 10% of adolescents, whereas snoring has been reported in 3.4-34% of elementary school children and severe sleep-disordered breathing in 1-4% of children.^9-11^ However, only 3.7% of children are diagnosed with obstructive sleep apnoea (OSA) in paediatric primary care centres, which is significantly lower than the prevalence of these disorders.^12^ Sleep problems in children are subject to variation, given the consideration of ethnic and sociocultural backgrounds. Scanty literature exists regarding sleep problems among children in Saudi Arabia as per BaHammam et al^13^ who found that sleep problems were prevalent among elementary school children in Saudi Arabia. Moreover, their study sheds light on sleep habits and practices in this age group in Saudi Arabia, such as the high prevalence of day-time napping.^13^ A large-sample examination of Saudi and non-Saudi nationals reported that sleep quality disturbance was notably augmented throughout the COVID-19 period. Approximately half of the 790 children enrolled in the study experienced a drastic change in sleeping habits.^14^ Worldwide, through the initiation of lockdown and home confinement measures upon the WHO declaration of COVID-19 as a worldwide pandemic, the sleep-wake cycle in children was altered in different ways. Italy was one of the first affected countries in Europe, which reported a significant delay in sleep habits exemplified by wake and bed-times, sleep duration, and sleep-wake cycle among children aged <13 years; adolescents were less impacted.^15^ In a large meta-analysis examining studies on the influence of COVID-19 on sleep, 5 were pooled as they were pertaining to the paediatric population and concluded an alarming change in meeting normal sleep recommendations.^16^ In a study examining bed-time anxiety alongside COVID-19-related anxiety at the start and end of lockdown, increased sleep latency was noted among United Kingdom children despite resilient sleep time. This effect abated once home confinement measures were lifted.^17^ Among Turkish children aged 6-12 years, bed-time resistance, sleep-onset delay, and sleep duration fell under the impact of the pandemic, with correlations noted between these disturbances and parental apprehension, helplessness and frightening during COVID-19.^18^ Thus, we were encouraged to lead the chance to examine the prevalence of sleep problems in a larger proportion of patients, given the new era changes affecting sleep patterns among children in Saudi Arabia pertaining to the COVID-19 era that has resulted in remarkable changes in lifestyle, as children nowadays use gadgets for long periods, accompanied by unhealthy eating habits in the presence of fast food and increasing obesity rates.

Sleep-disordered breathing is a clinical spectrum with frequent episodes of either partial or complete obstruction of the upper airway during sleep, ranging from snoring to apnoea. Breathing sleep disorders are classified, with increasing severity, as follows: primary snoring, upper airway respiratory resistance syndrome, obstructive hypoventilation, and OSA.^1^

Sleep pattern include parasomnias, night terror, nightmare, bruxism, enuresis, sleep walking, sleep talking, nap, and the International Classification of Sleep Disorders (ICSD) definition.^1^

Sleep problems include sleep onset association disorder, rhythmic movement disorder, limit setting sleep disorder/bedtime resistance, insufficient sleep syndrome, delayed sleep phase disorder, advanced sleep phase disorder, behavioural insomnia of childhood, and restless legs syndrome.^1^

## Methods

This cross-sectional study was carried out in King Fahad Medical City, Suliaman AlHabib Hospital, Prince Sultan Military Medical city, and among the public in Riyadh, Saudi Arabia, which has a population of more than 6 million. Boys and girls attending regular public elementary schools in all grades were eligible for this study. The data were obtained via an online questionnaire inquiring on demographic data, specific sleep problems, habits, and home environment based on previous studies, primarily BaHammam et al^13^ study, was distributed and completed by the parents or guardians. using Google forms when parents were encountered either at clinics, upon admission or through social media platforms (Twitter or WhatsApp).

International Review Boards approval (no.: RC22006-03) was obtained from Dr. Sulaiman AlHabib of Medical Center, Riyadh, Saudi Arabia.

The study duration was 2 months, from August through September 2022, and the sample size was calculated based on the incidence of paediatric sleep disorders of around 1-4%, whereas the prevalence of paediatric sleep problems was approximately 25%. Given an estimated Saudi Arabian population of 28,000,000, confidence interval of 95% and margin error of 5%, the minimum sample number was estimated to be 385. Children aged >2 and <14 years were enrolled in the study, whereas those aged <2 years, >14 years, or with chronic illness (such as congenital heart disease, sickle cell disease, chronic lung disease, prematurity, Down syndrome, and Prader-Willi syndrome) were excluded.

### Statistical analysis

Data was analyzed using the Statistical Package for the Social Sciences, version 20.0 (IBM Corp., Armonk, NY, USA). Pearson Chi-square and Fisher’s exact appropriate tests and Likelihood Ratio were used. Chi statistics were applied to measure variable independence. A *p*-value of <0.05 was considered significant.

## Results

The total sample of 585 participants included 345 (59%) boys and 240 (41%) girls. The mean age was 7 (range: 2-14) years, and 91% were Saudi. Participants’ demographics are presented in Table 1. The majority of children (70.2%) resisted sleep regardless of age (*p*=0.292), followed by sleep-onset delay (58.1%), difficulty waking up in the morning during weekdays (41.3%), weekends (38%), and interrupted sleep (31%; Table 2).

**Table 1:** - Demographics.

Characteristics	n (%)
***Gender***	
Male	345 (58.9)
Female	241 (41.1)
***Age group (years)***	
2-5	207 (35.3)
6-9	195 (33.3)
10-14	207 (31.4)
***Nationality***	
Saudi	533 (91.0)
Non-Saudi	53 (9.0)

Values are presented as numbers and precentages (%).

**Table 2:** - Sleep problems prevalence.

Sleep problems	n (%)
Bed-time resistance	411 (70.1)
Sleep-onset delay	340 (58.0)
Fear at bed-time	181 (30.9)
Interrupted sleep	187 (31.9)
Nightmares	156 (26.6)
Night terrors	121 (20.6)
Nocturnal enuresis	97 (16.6)
Sleep talking	167 (28.5)
Sleep walking	20 (3.4)
Difficulty waking up in the morning (WD)	241 (41.1)
Difficulty waking up in the morning (WE)	218 (37.2)
Snoring	87 (14.8)
Witnessed apnoea	23 (3.9)
Daytime napping	59 (10.1)
Sleep in class	52 (11.8)
My child is not getting enough sleep	153 (26.1)
Daytime fatigue	154 (26.3)
Hyperactivity	245 (41.8)
Inability to concentrate	175 (29.9)
My child is aggressive during the day	247 (42.2)
Co-sleeping with parents	210 (35.8)

Values are presented as numbers and precentages (%). WD: weekdays, WE: weekend

No statistical significance was noted between age group and difficulty falling asleep (*p*=0.121). The majority of children (69.5%), regardless of age, did not have feelings of fear associated with sleep (*p*<0.001). Moreover, 394 out of 571 children did not suffer from interrupted sleep regardless of their age group (*p*=0.091). No significant statistical link was noted between age group and nightmares (*p*=0.089) and 422 out of 571 children reported having no nightmares. However, 43% of those who woke up afraid were aged between 2-5 years, but this remains statistically insignificant between age groups (*p*=0.092). Participants in various age groups reported no statistical significance regarding experiencing nightmares. Nocturnal enuresis (*p*=0.693), sleep talking (*p*=0.957), and sleep walking (*p*=0.055) in relation to age were all statistically insignificant. Older children (aged 10-14 years) were more likely to have difficulty in waking up from sleep than younger children on all days of the week (*p*<0.001). Additionally, 44.7% of those who snored while sleeping were aged 10-14 years (*p*=0.025; Table 3).

**Table 3:** - Association between sleep problems and age groups.

Associated variables	Age groups (years)			*P*-values
	2-5	6-9	10-14	
Bed-time resistance	139 (34.7)	140 (34.9)	122 (30.4)	0.292
Sleep-onset delay	105 (31.6)	119 (35.8)	108 (32.5)	0.121
Fear at bed-time	52 (29.9)	81 (46.6)	41 (23.6)	0.000
Interrupted sleep	72 (40.7)	49 (27.7)	56 (31.6)	0.091
Nightmares	57 (38.3)	55 (36.9)	37 (24.8)	0.089
Night terrors	50 (43.1)	36 (31.0)	30 (25.9)	0.092
Nocturnal enuresis	37 (38.1)	32 (33.0)	28 (28.9)	0.693
Sleep talking	55 (34.0)	54 (33.3)	53 (32.7)	0.957
Sleep walking	3 (15.0)	6 (30.0)	11 (55.0)	0.055
Difficulty waking up in the morning (WD)	34 (14.4)	93 (39.4)	109 (46.2)	0.000
Difficulty waking up in the morning (WE)	40 (18.4)	77 (35.5)	100 (46.1)	0.000
Snoring	24 (28.2)	23 (27.1)	38 (44.7)	0.025
Witnessed apnoea	7 (30.4)	6 (26.1)	10 (43.5)	0.480
My child is not getting enough sleep	35 (23.8)	45 (30.6)	67 (45.6)	0.000
Co-sleeping with parents	28 (62.1)	56 (27.2)	22 (10.7)	0.000

Values are presented as numbers and precentages (%). WD: weekdays, WE: weekend

A total of 424 out of 571 children reported getting enough sleep regardless of age (*p*<0.001). Approximately 62.1% of those who were found to co-sleep with their parents were aged between 2-5 years (*p*<0.001). Interestingly, across all age groups, most children did not resist sleep when co-sleeping with their parents (*p*<0.001; Tables 3 & 4). The majority of children (408/571) resisted sleep when using screens before bed-time (*p*=0.002). Those aged >5 years had more than 2 hours of screen time before bed than younger children (*p*<0.001; Table 4).

**Table 4:** - Association between bed-time resistance and sleep habits with age group.

Associated variables	Age groups (years)			*P*-values
	2-5	6-9	10-14	
***My child resists sleep when***:				
Sleeping regularly	70 (33.7)	69 (33.2)	69 (33.2)	0.876
Co-sleeping with parents	65 (53.7)	32 (26.4)	24 (19.8)	0.000
Using gadgets (smartphones, tablets, TV, etc.)	126 (30.9)	137 (33.6)	145 (35.5)	0.002
Using gadgets (smartphones, tablets, TV, etc.) for <2 hours	94 (43.3)	71 (32.7)	52 (24.0)	0.000
Using gadgets (smartphones, tablets, TV, etc.) for >2 hours	52 (20.5)	91 (35.8)	111 (43.7)	0.000
Consuming caffeinated or energy drinks (coffee, tea, soda, etc.)	51 (29.3)	48 (27.6)	75 (43.1)	0.001
Lights on	112 (37.5)	92 (30.8)	95 (31.8)	0.322
Consuming late dinner	37 (27.4)	48 (35.6)	50 (37.0)	0.105
Napping during day-time	162 (35.2)	149 (32.4)	149 (32.4)	0.765

Values are presented as numbers and precentages (%).

Although the majority reported resistance to sleep at night with concomitant day-time napping, this correlation was not statistically significant (*p*=0.765). However, a positive correlation was found between day-time napping due to long school hours and early school days (*p*<0.001; Table 4).

Consuming stimulating drinks (energy drinks, soft drinks, coffee, and tea) and a late time dinner were neither affecting the children’s resistance to sleep nor caused difficulties in sleeping. To detail it, only 69% of kids have no bedtime resistance when consuming caffeinated drinks nor difficulties in sleeping as 76% consumed stimulating drinks with no resultant difficulties. Approximately 67% of children have no bedtime resistance when having a late dinner, on the other hand, 76% have no difficulty sleeping when late time meal is consumed. However, this correlation was statistically significant in older patients in the age group of 10-14 years who seemed to resist sleep and experienced a sleep-onset delay when consuming a late dinner (*p*<0.001) or energising drink (*p*<0.05; Tables 4 & 5).

**Table 5:** - Association between sleep-onset delay and sleep habits with age group.

Associated variables	Age groups (years)			*P*-values
	2-5	6-9	10-14	
***My child has difficulty entering sleep when***:				
Sleeping regularly	54 (34.4)	47 (29.9)	56 (35.7)	0.460
Co-sleeping with parents	52 (46.8)	30 (27.0)	29 (26.1)	0.013
Using gadgets (smartphones, tablets, TV, etc.)	110 (28.8)	131 (34.3)	141 (36.9)	0.000
Using gadgets (smartphones, tablets, TV, etc.) for <2 hours	86 (41.3)	72 (34.6)	50 (24.0)	0.000
Using gadgets (smartphones, tablets, TV, etc.) for >2 hours	54 (21.5)	85 (33.9)	112 (44.6)	0.000
Consuming caffeinated or energy drinks (coffee, tea, soda, etc.)	54 (29.0)	56 (30.1)	76 (40.9)	0.006
Lights on	106 (35.5)	97 (32.4)	96 (32.1)	0.929
Consuming late dinner	38 (27.7)	45 (32.8)	54 (39.4)	0.059
Napping during daytime	146 (34.5)	145 (34.3)	132 (31.2)	0.577

Values are presented as numbers and precentages (%).

More than half (382/571) of the participating children had difficulty entering sleep with the use of screens (*p*<0.001). However, co-sleeping with parents was associated with no difficulty in entering sleep (*p*=0.013; Table 5).

Snoring was significantly associated with difficulty waking from sleep on weekends (*p*=0.005), weekdays (*p*=0.001), frequent dozing during the day (*p*=0.004), struggling to stay awake during classes (*p*=0.003), day-time fatiguability (*p*=0.013), inability to concentrate (*p*<0.001), aggressiveness/agitation (*p*=0.004), and academic performance on average (*p*<0.000). Witnessed sleep apnoea was significantly correlated with difficulty in waking up from sleep on weekends (*p*=0.021), weekdays (*p*=0.018), struggling to stay awake during classes (*p*=0.008), hyperactivity during the day (*p*=0.001), inability to concentrate (*p*=0.019), frequent dozing during the day (*p*=0.001), and average academic performance (*p*<0.016; Table 6). In the presence of a family history of sleep disorders, no significant association was noted with sleep problems or habits across all age groups (*p*<0.147).

**Table 6:** - Association between sleep problems and sleep habits.

Associated variables	Sleep problems			
	Snoring	*P*-values	Witnessed apnoea	*P*-values
Difficulty waking from sleep during weekdays	49 (20.8)	0.001	15 (6.4)	0.018
Difficulty waking from sleep during weekends	44 (20.3)	0.005	14 (6.5)	0.021
Frequent dosing during the day	16 (27.6)	0.004	7 (12.1)	0.001
My child struggles during classes	16 (30.8)	0.003	6 (11.5)	0.008
My child is not getting enough sleep	56 (13.2)	0.056	16 (3.8)	0.599
Daytime fatigue	32 (21.1)	0.013	9 (5.9)	0.166
Hyperactivity	42 (17.6)	0.126	17 (7.1)	0.001
Inability to concentrate	42 (24.4)	0.000	12 (7.0)	0.019
My child is aggressive during the day	48 (19.8)	0.004	13 (5.4)	0.161

Values are presented as numbers and precentages (%).

## Discussion

Sleep disturbances are an important problem among the paediatric population because of their influence on children’s health and strong correlations with behavioural problems.^18^ We found significant associations in this study and estimated some of the commonly encountered sleep problems among children from Saudi Arabia in the second data document at the national level addressing these problems since 2006, when BaHammam et al^13^ reported their first data on the matter. Our study demonstrated bed-time resistance (71.3%) as the most prevalent sleep problem, followed by sleep-onset delay (58.1%), difficulty rising in the morning across all days of the week (41.3%), and interrupted sleep pattern (31%). These findings are consistent with those reported by Fadime et al^18^ who found that bedtime resistance is the most common sleep problem of around 48.1% and this was sided by BaHammam et al^13^ who reported bed-time resistance of 26.2%. However, our study proved that these problems have increased in magnitude in comparison to the previously mentioned studies. A favourable explanation could be the drastic change in lifestyle influenced by technological advancement.

We remarked that age as a single factor was not associated with increased disturbed sleep habits or problems, including difficulty in falling asleep, nocturnal enuresis, sleep talking/walking, or nightmares. However, certain habits of co-sleeping with parents were found to be common in the younger age group (2-5 years). However, older children were less likely to have sleep problems, which may reflect their lower reporting to parents. With respect to other studies addressing this relationship, Fadime et al^18^ reported no correlation between age group and sleep problems, which is consistent with our findings. In contrast, Oliviero et al^15^ reported that falling asleep difficulties, anxiety at bed-time, nightmares, and sleep terrors were evident in the younger age groups. This supports the hypothesis that younger children are more prone to be afraid and prefer co-sleeping with parents to calm down, the prevalence of which was high in our sample (41%) in children aged 2-5 years. The prevalence of nocturnal enuresis was approximately 17% in our data, which is considered high compared with that reported by BaHammam et al^13^ (12.2%) and across the literature of 2-10%.^19-24^ A previously published study in the eastern region of Saudi Arabia by Kalo et al^25^ reported a prevalence of 16.3% among boys and 13.8% among girls. This variation can be explained by different sample sizes and questioning methods, in addition to the cultural background among all study samples. Day-time napping was highly prevalent in our sample (69.2%) and mostly attributed to early school timing (43.6%). BaHammam et al^13^ reported nappers to be around 40.8%, which has remained high throughout the last 2 decades despite the drastic lifestyle changes such as introduction of laptops, tablets, smartphones, and online classes that are more common nowadays, especially since COVID-19 lockdown. This finding of COVID-19 confinement and online schooling affecting day-time energy was supported by the findings of Oliviero et al.^15^ Moreover, the majority of children (71.5%) in our sample were found to resist sleep when using screens before bed-time, particularly those aged >5 years who had more than 2 hours’ worth of screen time before bed than younger children. This could possibly add up to sleep deprivation, affecting their day-time energy. Interestingly, our data demonstrated that the older age group (10-14 years) had more sleep disturbances in relation to consumption of late dinner or energising drinks than those aged <10 years. This can be explained by parental control over the eating and drinking habits of younger children, which is not the case in their older counterparts. These findings concur with those of Hogenkamp et al^21^ and Fadime et al^18^ who described the effect of consuming stimulants and late meals on adolescents.

In our study, snoring was reported in 14.9% of children compared with 17% reported by BaHammam et al^13^ and even less than that reported in the literature (3.6%).^18^ Snoring and witnessed apnoea were both proven to be significantly associated with disturbed sleep habits, including day-time fatiguability, inability to concentrate, aggression and hyperactivity, an academic performance of ‘Average’, and difficulty in risetime particularly in older age group (10-14 years) in our data. However, Fadime et al^18^ reported snoring as common among the younger age groups (4-5 years). Collectively, this has been confirmed and established clinically, which is in line with the known potential development of OSA in the background of a snoring child.

### Study strengths & limitations

Its cross-sectional design and short study duration without seasonal change. Also, this sample despite the great response rate and its conductance in the capital city of Riyadh, Saudi Arabia, it does not reflect or represent the whole Kingdom. However, on the other hand, strengths of our data lie on the fact that it is the second document on paediatric sleep habits and problems in Saudi Arabia to BaHammam et al.^13^ We also reported very alarming numbers that are worse than those reported earlier by BaHammam et al.^13^ Our questions were re-designed to match recent time changes as technological revolution.

In conclusion, this is a re-visit of what BaHamam et al^13^ have reported in 2006 concerning sleep habits and problems among children. The study sheds some light on sleep habits and practices in this age group in Saudi Arabia, such as the high prevalence of bed-time resistance, sleep-onset delay, hyperactivity, and sleep-affecting culprits such as screen time, snoring, and witnessed apnoea through the new era changes reflecting differences from the reported first document on this matter. Thus, sleep is an important research area in Saudi Arabia, and we believe our study would help guide further projects on paediatric sleep patterns across a larger scale.
